# An Insight to How Estate Women in Sri Lanka Manage Abnormal Vaginal Discharge

**Published:** 2017-02

**Authors:** Ilankoon IMPS, Goonewardena CSE, Fernandopulle RC, Perera PPR

**Affiliations:** 1. Dept. of Allied Health Sciences, Faculty of Medical Sciences, University of Sri Jayewardenepura, Nugegoda, Sri Lanka; 2. Dept. of Community Medicine, Faculty of Medical Sciences, University of Sri Jayewardenepura, Nugegoda, Sri Lanka; 3. Dept. of Obstetrics and Gynecology, Faculty of Medical Sciences, University of Sri Jayewardenepura, Nugegoda, Sri Lanka; 4. Dept. of Biochemistry, Faculty of Medical Sciences, University of Sri Jayewardenepura, Nugegoda, Sri Lanka

## Dear Editor-in-Chief

Women living in estate communities in Sri Lanka are vulnerable to many unhealthy practices due to the low socio-economical status, structural and social inequalities. Further, the presence of stigma and discrimination may discourage women from revealing their reproductive and sexual health issues and thereby increase the possibility of transmitting diseases. Abnormal vaginal discharge is a common gynaecological complaint among women in reproductive age. Persistent vaginal discharge can cause considerable distress to many women (1).

Physiological reasons for increased vaginal discharge are high estrogen levels during mid-cycle, pregnancy and due to sexual arousal. Pathological causes can be infective (Sexually Transmitted Infections (STIs) and non-STIs) and non-infective (genital tract malignancy, fistulae, allergic reactions and atrophic vaginitis associated with menopause) with the most common being the infective agents (2). Differentiating the normal and abnormal vaginal discharge is essential in early detection of pathological discharge in order to prevent the possible complications of delayed treatment for reproductive tract infections such as infertility, ectopic pregnancy, an increased risk of HIV transmission and infant death.

Many women use self-treatment with over-the-counter preparations for vaginal discharge prior to consulting a doctor due to many reasons (1). Thus, this descriptive qualitative study was aimed to assess how women living in estate communities in Colombo District, Sri Lanka manage abnormal vaginal discharge, including their cultural practices and health seeking behaviors. Three Focus Group Discussions (FGDs) were conducted in August 2015 in two estates and all groups comprised of six to ten women with a total of 20 women.

Majority of the study sample consisted of Indian Tamils (n=17, 85%) and the predominant religion was Hindu (n=15, 75%). Most were educated up to grade 11 (65%, n=13) i.e. compatible with secondary sophomore in the US, but there were 30% with just primary education. Most of the women have had a previous history of vaginal discharge and they perceived it as a usual or normal condition. Transcribed verbatim data were analyzed using qualitative content analysis and six themes related to vaginal discharge concerns emerged namely difficulty in differentiating normal from abnormal vaginal discharge, lack of knowledge on causative factors, cultural influences, and beliefs, unstable/ limited source of income, fear of disclosing and lack of support system.

Majority of women living in estate communities work in rubber estates full time for earning and they find difficulties in accessing additional health information. They had poor knowledge and health seeking behaviors related to vaginal discharge. Similarly, most of them had ignored their health problems due to different factors like home responsibilities, domestic chores, children, and excessive burden of work at home (3). Women have difficulty in differentiating normal from abnormal vaginal discharge. Lack of awareness was a theme described in another study (3). Majority of the participants ignored this problem, and thus they did not share it with another person (3).

In the present study, women perceived body heat, STIs, heavy work and consumptions of heaty food as the causes for excessive vaginal discharge while some other studies found melting bones (3), existing pressures of poverty and everyday pressures (4), infectious causes (5) as causative factors indicating the different cultural beliefs. Further women have used different home remedies such as “polpala” *(Aerva lanata)* herbal drink or “Neeramulliya” *(Asteracantha longifolia Linn)* herbal drink, king coconut, Sauw *(sago)* Kanji, Uluhal *(Fenugreek) and Aloe vera* juice in order to manage vaginal discharge in the present estate community. These self-remedies were tried out by this community because of difficulty in disclosing the condition to a doctor, due to perceived stigma and fear of examination and hospitalization. Similarly, women were reluctant to discuss vaginal symptoms even with their physicians, worrying they will be seen as sexually promiscuous (5). Further, some women mentioned about difficulty in accessing health care, as they are busy with their employment and household work in the present community. Further avoidance of wearing tight trousers, hot baths, washing perineal area frequently, use of salt baths and use of natural yogurt (6) and over-the-counter medicines (1, 6) have been found to be practiced in other communities. A more typical pattern was to seek medical consultation after a variety of other options had been tried and had failed (5) which were similar to the findings of this study.

Women living in estate communities in Sri Lanka have culturally sensitive practices and poor health seeking behavior related to abnormal vaginal discharge. This shows the importance of planning health education interventions concerning the sociocultural context to improve their health seeking behaviors. Women had fear of disclosing health matters related to reproductive and sexual health due to possible stigma and discrimination from the society. Therefore, providing additional health support services in order to prevent transmission of STIs and to prevent unhealthy gynecological practices is essential.
